# Phytochemical Characterization and High-Speed Countercurrent Chromatography-Assisted Isolation of Flavonoids from Leaves of *Talisia esculenta* Radlk

**DOI:** 10.3390/plants15142229

**Published:** 2026-07-21

**Authors:** Katia Castanho Scortecci, Letícia Barbosa Santos, Luís Felipe Costa Gatis, Verônica Giuliani de Queiroz Aquino-Martins, Nathalia Maira Cabral de Medeiros, Wirginia Kukula-Koch, Sarah Stiegeler, Garance Coquant, Fabio Boylan, Sinéad C. Corr

**Affiliations:** 1 School of Pharmacy and Pharmaceutical Sciences, Trinity Biomedical Sciences Institute, Trinity College Dublin, D02 R590 Dublin, Ireland; kacscort@yahoo.com (K.C.S.); leticiabsantos@ymail.com (L.B.S.); 2 Laboratório de Transformação de Plantas e Análise em Microscopia (LTPAM), Departamento de Biologia Celular e Genética, Universidade Federal do Rio Grande do Norte (UFRN), Natal 59078-970, Brazil; luis.costa.099@ufrn.edu.br (L.F.C.G.); veronica.aquino@ufrn.br (V.G.d.Q.A.-M.); nathaliamaira@gmail.com (N.M.C.d.M.); 3 Programa de Pós-Graduação em Bioquímica e Biologia Molecular, Centro de Biociências, Universidade Federal do Rio Grande do Norte, Natal 59078-970, Brazil; 4 School of Microbiology and APC Microbiome Ireland, University College Cork, T12 YT20 Cork, Irelandgarance.coquant@ucc.ie (G.C.); scorr@ucc.ie (S.C.C.); 5 Pharmaceutical Products Department, College of Pharmacy, Federal University of Minas Gerais, Belo Horizonte 31270-901, Brazil; 6 Department of Pharmacognosy with Medicinal Plants Garden, Medical University of Lublin, 20-093 Lublin, Poland; virginia.kukula@gmail.com; 7 Trinity Natural Products Research Centre, NatPro Centre, Trinity College Dublin, D02 PN40 Dublin, Ireland

**Keywords:** secondary metabolism, phenolic compounds, glycosylated flavonols, tropical tree species, bioactive constituents

## Abstract

*Talisia esculenta* Radlk (Sapindaceae) is a tropical tree species native to Brazil, whose leaves are traditionally consumed as herbal infusions. However, the secondary metabolite composition of its foliage remains poorly characterised. In this study, the phytochemical profile of *T. esculenta* leaf infusions was investigated using HPLC–ESI–QTOF–MS/MS, leading to the annotation of 20 compounds. Subsequent liquid–liquid partitioning and high-speed counter-current chromatography (HSCCC) enabled the targeted isolation of two major constituents. The analysis revealed a flavonoid-rich profile dominated by glycosylated flavonols, with rutin and quercitrin identified as the principal compounds and structurally confirmed by NMR spectroscopy. They were both quantified in the aqueous infusion of the leaves, being 1.42 ± 0.02% (*w*/*w*) and 1.54 ± 0.01% (*w*/*w*) for rutin and quercitrin, respectively. The metabolite pattern found is consistent with that reported in other members of the Sapindaceae family and contributes to the chemotaxonomic characterisation of the genus. Fractionation enhanced the enrichment of phenolic constituents and allowed assessment of their functional properties such as antioxidant activity (including DPPH assay: from 43.4% for the aqueous infusion to 92.9% for the ethyl acetate fraction) and inhibition of microbial biofilm formation (from 37.4% for the aqueous infusion to 83.4% for the butanolic fraction at 6.25 µg/mL against *Staphylococcus aureus*). The MIC and MBC were also calculated for the aqueous infusion (12.5 mg/mL for both), its partitions as well as rutin and quercitrin. Anti-inflammatory effects were also observed, with inhibition of NF-κB activity reaching 56.9% for the aqueous infusion and 47.7% for the butanolic fraction. These findings expand current knowledge of secondary metabolite diversity in *T. esculenta* and demonstrate the applicability of HSCCC as an efficient technique for flavonoid isolation from complex plant extracts. Overall, this study provides new insights into the phytochemical composition of an underexplored tropical species within the Sapindaceae family.

## 1. Introduction

Despite advances in modern medicine, plants remain an important source of bioactive compounds and functional extracts, particularly in preventive nutrition and the development of plant-based ingredients. Research on medicinal and edible plants continues to contribute to the characterisation of biologically active constituents and their associated properties, including antioxidant and immunomodulatory effects [1,2]. Among plant secondary metabolites, phenolic compounds, especially flavonoids, have attracted considerable attention due to their widespread occurrence in higher plants, well-established antioxidant activity, and ability to modulate cellular signalling pathways involved in oxidative stress and inflammation [3,4,5].

Against this backdrop of Brazil’s rich plant biodiversity, this study focuses on *T. esculenta* Radlk (Sapindaceae), commonly known as “pitombeira”. Native to Central and South America, this species has long been used in traditional medicine [6]. It produces edible fruits that are widely consumed and traded in local markets, often regarded as exotic produce. In addition to its nutritional value, the leaves are used in Brazilian folk medicine, particularly as aqueous infusions for the treatment of inflammatory conditions such as rheumatism [7]. However, the chemical composition of the leaves remains insufficiently explored, and their pharmacological properties have not been fully validated [7,8].

Previous studies have reported phenolic compounds, including flavonoids, and other bioactive constituents in extracts from various plant organs, suggesting antioxidant, antimicrobial, and anti-inflammatory potential [8,9]. Nevertheless, compared with other members of the Sapindaceae family, the phytochemical profile of *T. esculenta* leaves remains under-characterised, and the biological activities of individual constituents are still poorly understood. Moreover, despite promising preclinical findings, clinical evidence supporting its therapeutic use in humans remains limited.

Given these gaps, further studies are required to identify the major bioactive constituents of *T. esculenta* leaves and to evaluate their biological potential. Accordingly, the present work aimed to investigate the phytochemical composition and bioactivity of *T. esculenta* leaf extracts, with emphasis on the characterisation of bioactive fractions and isolation of major compounds. Fractionation followed by high-speed countercurrent chromatography (HSCCC) enabled the isolation of rutin and quercitrin as the predominant flavonoids.

HSCCC is a support-free liquid–liquid chromatographic technique that separates compounds according to their partition coefficients (K values) between two immiscible liquid phases retained under centrifugal force. Unlike conventional solid-phase chromatography, HSCCC eliminates irreversible adsorption of analytes to a stationary support, thereby enabling high recovery, excellent sample loading capacity and efficient isolation of labile or highly adsorptive natural products. These characteristics make HSCCC particularly well suited for the preparative fractionation of complex botanical extracts, where preservation of compound integrity and recovery are essential. These features have made HSCCC an established method for the preparative purification of flavonoids from complex botanical extracts prior to structural elucidation and biological evaluation [9].

The antioxidant, antimicrobial, and anti-inflammatory activities of the fractions and isolated compounds (when possible, due to the amount isolated) were evaluated using in vitro assays, including DPPH, total phenolics, total antioxidant capacity, copper chelation, inhibition of *Staphylococcus aureus* biofilm formation and modulation of NF-κB activity in the RAW-Blue™ macrophage reporter cell line. Overall, the results demonstrate antioxidant activity and moderate antimicrobial potential in *T. esculenta* leaf-derived fractions and flavonoids.

## 2. Results

### 2.1. Assignment of Low-Molecular-Weight Compounds by HPLC-ESI-QTOF-MS/MS

**Figure 1:**
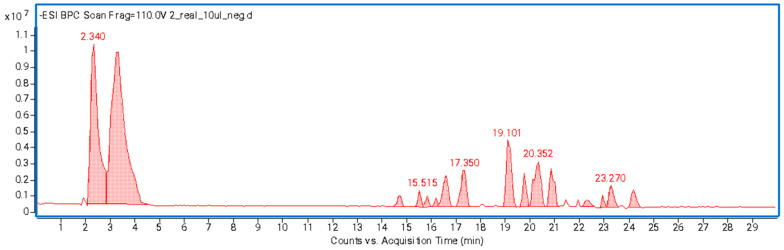
Mass chromatogram of the investigated *Talisia esculenta* extract in the negative ion mode.

The aim of this study was to characterise the phytochemical profile of *T. esculenta* leaf infusion using LC–MS and LC–MS/MS. Initially, the mass spectrometric parameters and solvent gradient were optimised to obtain high-quality spectra and enable reliable annotation of individual constituents (Figure 1).

The tentative identification of low-molecular-weight compounds was achieved based on accurate mass measurements, fragmentation pattern analysis, comparison with mass spectral databases (METLIN and HMDB) and previously published literature. The analysis confirmed the presence of a broad range of phenolic compounds, including phenolic acids, flavonoids, and flavonoid glycosides, within a mass deviation tolerance of 10 ppm (Table 1 and Table S1, Figure S1).

Several compounds previously reported in *T. esculenta* extracts were detected, including catechin, epicatechin, rutin, and quercetin rhamnoside [10,11]. However, simple phenolic acids such as gallic, syringic, ferulic, and caffeic acids were not observed. Instead, protocatechuic acid was identified in its free form, while most phenolic acids occurred as conjugated derivatives, including glucuronides and sugar esters. Glucogallic acid was tentatively annotated at approximately 11 min (*m/z* 331), based on its characteristic fragment ion at *m/z* 168. Caffeoyl–glucuronic acid derivatives were also putatively identified, showing signals at *m/z* 355 and fragmentation patterns consistent with the loss of caffeic acid and a hexose moiety. These results indicate that phenolic acids are predominantly present as conjugated forms rather than as free acids.

A prominent signal at *m/z* 431, corresponding to luteolin hexoside, was observed, with a characteristic fragment ion at *m/z* 284. Luteolin glucosides have previously been reported in the fruit peel of this species by Alves et al. [12]. Additional compounds detected included procyanidin B isomers (*m/z* 577), luteolin hexoside–deoxyhexoside (*m/z* 593), and methoxy-quercetin-O-hexoside–deoxyhexoside (*m/z* 623). Overall, the metabolite profile was consistent with that reported for other Sapindaceae species [13]. A strong signal corresponding to citric acid was also detected at the start of the chromatogram, indicating its high abundance [14].

Quantitative LC–MS analysis confirmed the presence of both rutin and quercitrin in the *T. esculenta* extract, with concentrations consistently above the limits of detection and quantification (see Figure S1 and Table S2 in the Supplementary Information). Calibration curves for both standards showed good linearity over the tested concentration range (0.001–0.1 mg/mL), demonstrating the suitability of the method for quantitative analysis. The calibration equations were as follows: y = 2,750,819,408.1056x + 10,122,651.6786 (R^2^ = 0.9974) for quercitrin, and y = 3,438,709,610.5561x + 33,102,118.8605 (R^2^ = 0.9972) for rutin. Linearity was maintained across the analytical range, with no significant deviations from regression observed.

Based on these data, the rutin content was calculated as 1.42 ± 0.02% (*w*/*w*). The low standard deviation indicates good analytical reproducibility. For quercitrin, the mean content was 1.54 ± 0.01% (*w*/*w*), likewise showing low variability across samples. Overall, both flavonoids were present at comparable concentrations, indicating that *T. esculenta* leaf infusion is a consistent and relatively rich source of these bioactive compounds.

### 2.2. HSCCC Separation

The aqueous infusion of *T. esculenta* leaves was sequentially partitioned with ethyl acetate (EA) and *n*-butanol (B) to enrich flavonoid-containing fractions prior to HSCCC separation. Thin-layer chromatography (TLC) conditions were first optimised by evaluating several solvent systems. The best resolution was achieved using ethyl acetate:formic acid:acetic acid:water (10:0.5:0.5:0.5, *v*/*v*/*v*/*v*). Visualisation with Natural Products/Polyethylene Glycol (NP/PEG) reagent revealed orange–yellow bands characteristic of flavonoids, alongside blue bands indicative of phenolic compounds.

**Figure 2:**
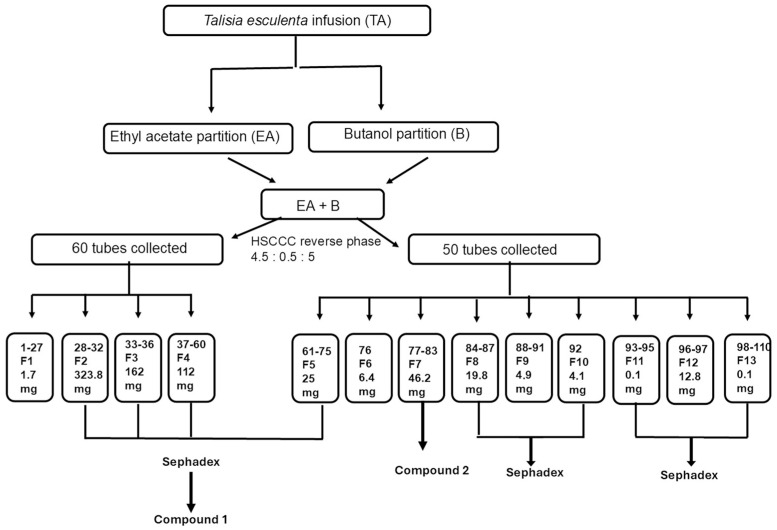
*Talisia esculenta* High Speed Countercurrent Chromatography fractions and the isolation of two flavonoid compounds.

**Figure 3:**
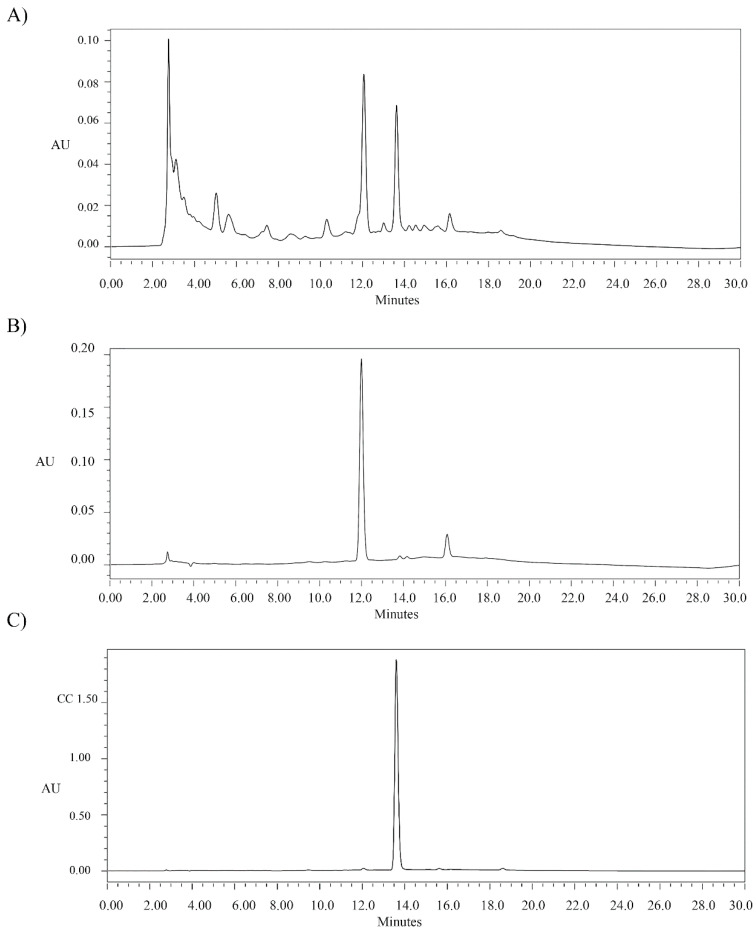
HPLC analysis of two flavonoids isolated from HSCCC. In (**A**), the chromatogram from aqueous infusion is depicted (AI), while (**B**) depicts the one obtained from the isolated rutin (F2—compound **1**), and (**C**) the one from the isolated quercitrin (F7—compound **2**).

For HSCCC, multiple biphasic solvent systems were tested. The optimal system was ethyl acetate:*n*-butanol:water (4.5:0.5:5, *v*/*v*/*v*), which provided suitable partitioning behaviour. As the EA and B fractions showed comparable TLC profiles, they were combined prior to HSCCC separation. The separation was performed in reverse mode, yielding 110 fractions that were pooled into 13 groups based on TLC similarity (Figure 2). Compound **2** was isolated from HSCCC fractions, while the second major compound was obtained after further purification using Sephadex LH-20. These compounds were identified by NMR as rutin (compound **1**) and quercitrin (compound **2**) (Figure 2) [15,16], and their purity was confirmed by HPLC–UV analysis (Figure 3).

### 2.3. Antioxidant Activity

**Figure 4:**
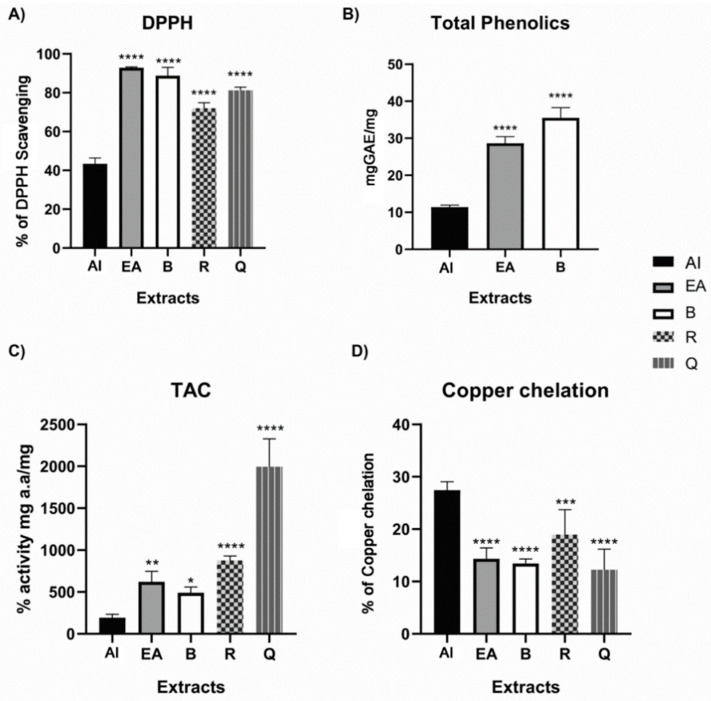
Antioxidant activity of *Talisia esculenta* partitions using in vitro assays. (**A**) scavenging of the DPPH radical, (**B**) total phenolics, (**C**) total antioxidant capacity (TAC) in leaves, (**D**) copper chelation. AI corresponds to leaf aqueous infusion, EA corresponds to the ethyl acetate partition, B corresponds to the *n*-butanol partition, R to rutin, Q to quercitrin, and GAE = Galic acid equivalent. These assays were performed in triplicate, and the experiment was repeated twice. The error bars represent SD (standard deviation), different asterisks represent statistically significant differences among treatments using the Tukey test: * (*p* < 0.05), ** (*p* < 0.01), *** (*p* < 0.001), and **** (*p* < 0.0001).

The antioxidant activity of the aqueous infusion (WI), ethyl acetate (EA), and *n*-butanol (B) fractions, as well as the isolated compounds, was assessed using several in vitro assays. In the DPPH assay, the EA and B fractions exhibited approximately twice the activity of the crude infusion, while rutin and quercitrin showed comparable effects (Figure 4A). Total phenolic content was higher in the EA and B fractions than in the crude extract (Figure 4B). Similarly, total antioxidant capacity (TAC) was significantly enhanced in the fractions, with the isolated compounds showing the highest activity (Figure 4C). Previous work by Cordeiro et al. [17] reported antioxidant activity for the leaf infusion. In contrast, copper chelation activity was greatest in the crude infusion, suggesting that synergistic interactions between constituents may contribute to this effect (Figure 4D).

### 2.4. Antimicrobial Activity

#### 2.4.1. Minimum Inhibitory Concentration and Minimum Bactericidal Concentration

**Figure 5:**
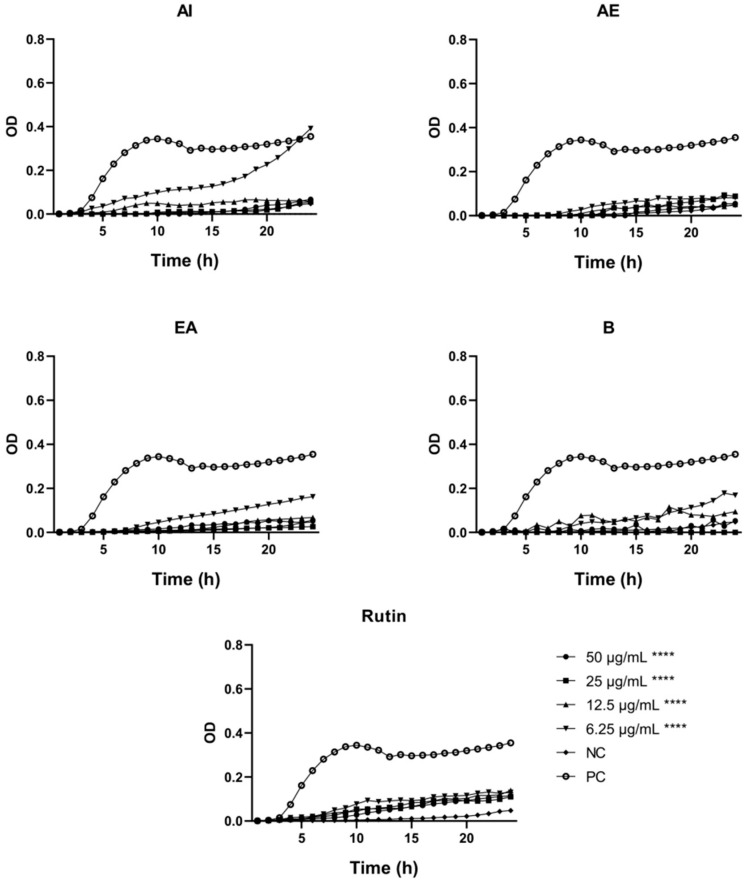
Growth kinetics assay over 24 h using *Talisia esculenta* extracts against *Staphylococcus aureus* NCTC 6571. AI corresponds to leaf aqueous infusion, AE corresponds to after extraction, EA corresponds to the ethyl acetate partition, and B corresponds to the *n*-butanol partition. Statistical significance was evaluated using Tukey’s post hoc test. The error bars represent SD (standard deviation), with significance levels denoted as follows: The Tukey assay was used for statistical analysis, where the asterisks represent: **** (*p* < 0.0001).

Antimicrobial activity was evaluated against five bacterial strains. The extracts showed selective antibacterial effects, with *Staphylococcus aureus* NCTC 6571 being the most susceptible. All samples inhibited *S. aureus*, with MIC and MBC values indicating bactericidal activity (Table 2). In contrast, *Escherichia coli*, *Pseudomonas aeruginosa*, *Listeria monocytogenes*, and *Salmonella enterica* were resistant even at higher concentrations. Growth kinetics assays confirmed concentration-dependent inhibition, particularly at higher extract doses, while partial regrowth at lower concentrations suggested a predominantly bacteriostatic effect under those conditions (Figure 5).

#### 2.4.2. Biofilm Formation

**Figure 6:**
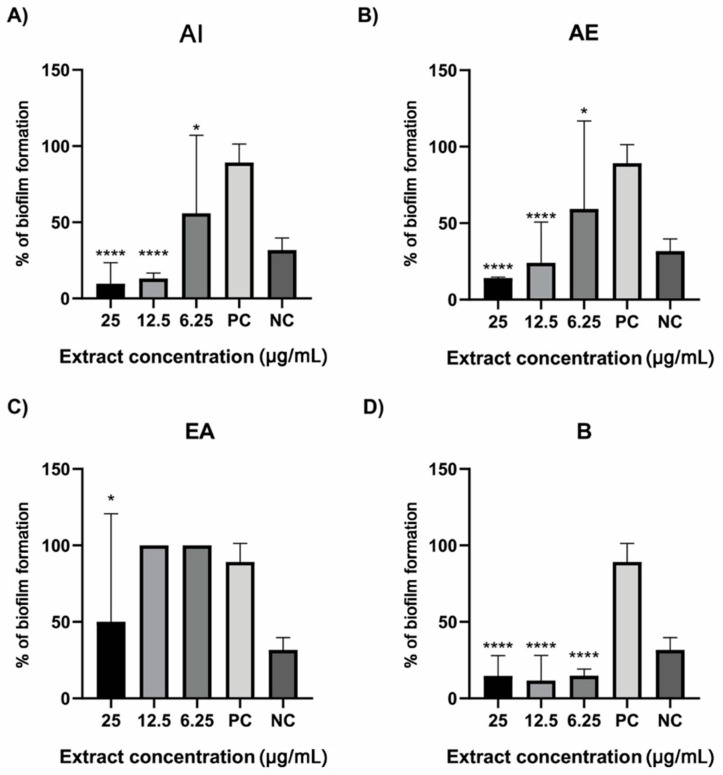
Effect of *Talisia esculenta* extract on biofilm formation using *Staphylococcus aureus* NTC NCTC 6571. PC corresponds to growth control (medium and bacteria), NC corresponds to sterile control (only medium). The *y*-axis represents the percentage value obtained from GC, with 100% as the maximum. The *x*-axis indicates the concentrations of the compound tested. The graph displays data compiled from four independent experiments. (**A**) AI corresponds to leaf aqueous infusion, (**B**) AE corresponds to the after extraction, (**C**) EA corresponds to ethyl acetate partition. (**D**) B corresponds to *n*-butanol partition. Statistical analysis was performed using Tukey’s post hoc test, the error bars represent SD (standard deviation) with significance levels indicated as follows: where the asterisk represents * (*p* < 0.05) and **** (*p* < 0.0001).

All samples also reduced biofilm formation by *S. aureus*. The *n*-butanol fraction showed the strongest inhibition across most concentrations, whereas the ethyl acetate fraction was only effective at higher doses (Figure 6).

### 2.5. Molecular Docking

Molecular docking analysis indicated favourable binding interactions between rutin and quercitrin and key *S. aureus* surface proteins, including Surface Protein G, a biofilm-associated protein, and Clumping Factor B. Rutin showed stronger binding affinities and formed more hydrogen bonds than quercitrin, suggesting greater binding stability (Figures S2 and S3). These interactions were primarily located in regions involved in bacterial adhesion and biofilm formation.

### 2.6. Anti-Inflammatory Activity

**Figure 7:**
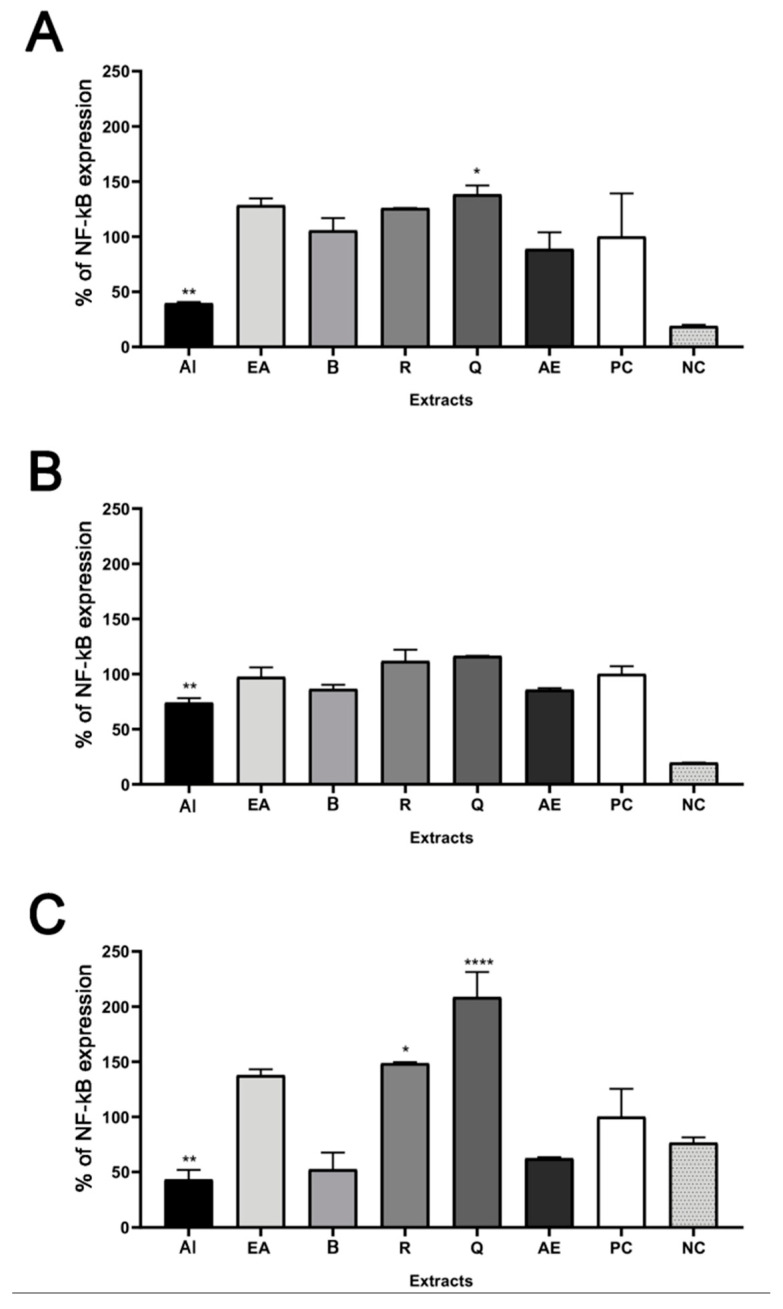
In vitro assays on RAW 264.7 macrophage cells. (**A**) shows the effect on NF-kB expression when samples and LPS were added simultaneously. (**B**) corresponds to the effect of the expression of NF-kB when the samples were added first, and then, after 5 h, the LPS. (**C**) It was the same, only that LPS was added after 10 h. The *y*-axis represents the percentage considering Positive Control (cells in the medium + LPS) as 100%. The concentration used was 250 µg/mL. NC (negative control) represents macrophages with culture medium only, and PC (positive control) represents macrophages treated with LPS in culture medium, with PC corresponding to 100% NF-kB expression. AI—Leaf aqueous infusion, EA—partition obtained with ethyl acetate, B—partition obtained with *n*-butanol, AE—extract obtained after these two partitions; Rutin and Quercitrin, the two compounds isolated. The assay was made in triplicate; statistical analysis was done using the Tukey test. The error bars represent SD (standard deviation) with significance levels indicated as follows: where the asterisk represents: * (*p* < 0.05), ** (*p* < 0.01) and **** (*p* < 0.0001).

The anti-inflammatory potential was assessed in RAW-Blue™ macrophages by measuring NF-κB activation. Both the crude extract and fractions reduced NF-κB activation at higher concentrations, particularly when administered prior to lipopolysaccharide (LPS) stimulation, suggesting a protective effect (Figure 7 and Figure S4). The *n*-butanol fraction exhibited the most pronounced activity.

## 3. Discussion

The present study expands current phytochemical knowledge of *T. esculenta* leaves by showing that the traditionally consumed aqueous infusion is characterised by a flavonoid-rich profile dominated by glycosylated flavonols. Importantly, analysing the infusion rather than organic solvent extracts provides a more realistic representation of the compounds to which consumers are actually exposed. This approach strengthens the ethnopharmacological relevance of the findings and enables a more direct link between traditional use and observed biological activity.

In contrast to previous reports describing simple phenolic acids in *T. esculenta* leaves, these compounds were not detected in their free form in the present study. Instead, conjugated derivatives, including protocatechuic- and caffeic acid-related compounds, were identified [10,11,18]. Such discrepancies are not unusual in phytochemical studies and may reflect differences in environmental conditions, geographical origin, seasonality, plant developmental stage, genetic variability, and extraction methodology [19,20]. In particular, hot-water extraction may preferentially recover polar glycosylated constituents while limiting the extraction or stability of certain free phenolic acids. These observations underline the importance of methodological consistency when comparing phytochemical profiles and suggest that the chemical composition of *T. esculenta* may be more variable than previously assumed.

The predominance of flavonoid glycosides, especially rutin and quercitrin, is consistent with the well-established role of flavonol derivatives in plant metabolism. These compounds are widely recognised for their strong antioxidant properties, including hydrogen-donating capacity, reactive oxygen species scavenging, and inhibition of oxidative chain reactions [2,18,21,22,23,24]. In addition to these effects, rutin and quercitrin have demonstrated antimicrobial, anti-inflammatory, and cytoprotective activities in both in vitro and in vivo models, suggesting that they likely contribute substantially to the biological activity of *T. esculenta* infusion. Their identification therefore provides a plausible chemical basis for the species’ traditional medicinal use.

Fractionation of the infusion led to an increase in antioxidant activity, particularly in flavonoid-enriched fractions, indicating effective concentration of bioactive constituents [25,26]. This may reflect the view that flavonoids are key contributors to the overall antioxidant capacity of *T. esculenta*. However, the observed reduction in copper-chelating activity following partitioning with ethyl acetate and *n*-butanol suggests a more complex mechanism underlying this effect. Metal chelation typically arises from the combined action of multiple phytochemicals, and fractionation may disrupt synergistic interactions present in the crude infusion [27]. This highlights an important principle in phytotherapy: whole extracts may exhibit biological activities that cannot be fully replicated by isolated fractions or single compounds. Such synergy may enhance efficacy through complementary mechanisms, including radical scavenging, metal binding, and modulation of redox-sensitive signalling pathways.

Antimicrobial assays demonstrated selective activity against *Staphylococcus aureus*, including inhibition of biofilm formation, which is clinically relevant given the role of biofilms in persistent infections. Biofilm development is a major virulence mechanism that increases bacterial resistance to antibiotics and host defences. The persistence of antibiofilm activity despite reduced copper-chelating capacity suggests that this effect is mediated through mechanisms other than metal sequestration. Instead, it is likely related to interference with bacterial adhesion, quorum sensing, extracellular matrix production, and other processes involved in biofilm formation. Similar mechanisms have been reported for flavonoid-rich plant extracts, supporting the biological relevance of the compounds identified here [28,29,30,31,32].

Molecular docking analyses provided further mechanistic insight, revealing favourable interactions between the major flavonoids and *S. aureus* surface-associated proteins involved in adhesion [28,29,30,31]. These findings are consistent with the hypothesis that flavonoids may inhibit early stages of bacterial colonisation by reducing microbial attachment and subsequent biofilm development [30,31,32]. This mode of action is particularly advantageous, as it targets virulence rather than bacterial viability, thereby potentially reducing selective pressure for antimicrobial resistance. However, docking studies are predictive in nature, and these interactions require experimental validation through approaches such as gene expression analysis, protein-binding assays, and in vitro adhesion models.

The anti-inflammatory activity observed for the aqueous infusion provides additional support for its traditional use in the treatment of rheumatism and other inflammatory conditions [7]. LC–MS analysis indicated a high abundance of phenolic compounds, which likely contribute to both the antioxidant and anti-inflammatory effects observed. Oxidative stress and inflammation are closely interconnected processes, with reactive oxygen species acting as key mediators of inflammatory signalling [2,17,18,33]. By reducing oxidative stress, phenolic compounds may indirectly attenuate inflammatory responses. Moreover, flavonoids such as rutin and quercetin derivatives are known to modulate key inflammatory pathways, including NF-κB, MAPK, and Nrf2 signalling. Inhibition of NF-κB activation reduces the expression of pro-inflammatory cytokines and mediators such as cyclooxygenase-2 and inducible nitric oxide synthase, thereby dampening the inflammatory response [2,18,33]. The effects observed in this study are therefore consistent with both the phytochemical profile of the infusion and established mechanisms of related phenolic compounds.

Overall, these results indicate that the biological activity of *T. esculenta* is multifactorial, arising from combined antioxidant, antimicrobial, antibiofilm, and anti-inflammatory mechanisms. While flavonoid-enriched fractions showed enhanced activity in specific assays, the superior performance of the crude infusion in others underscores the importance of phytochemical complexity and synergistic interactions among constituents. These findings provide evidence for the traditional use of *T. esculenta* and highlight its potential as a source of bioactive compounds for functional foods, phytotherapeutic applications, and adjunct strategies targeting oxidative stress, inflammation, and bacterial infections. Future work should focus on the isolation of additional active constituents, the investigation of synergistic effects, and the evaluation of bioavailability, efficacy, and safety in appropriate in vivo models.

## 4. Materials and Methods

### 4.1. Chemicals

An aqueous infusion of *T. esculenta* was prepared and partitioned using ethyl acetate and *n*-butanol (HPLC grade) obtained from Sigma-Aldrich (Arklow, Ireland). Ultra-pure water was obtained from a Millipore™ system (Sigma-Aldrich, Arklow, Ireland). Sephadex^®^ LH-20, methanol for Sephadex separation, and methanol-d4 (≥99.8% purity) used for nuclear magnetic resonance (NMR) analysis were also purchased from Sigma-Aldrich (Arklow, Ireland).

### 4.2. Plant Material

Leaves of T. esculenta were collected in June 2023 in Parnamirim, Rio Grande do Norte, Brazil (−05°47′42″, +35°12′34″). Collection was authorised by the Biodiversity Authorisation and Information System (SISBIO), Brazil (licence registration no. 70956, SIGEN A39FD4C). The species was identified by Dr Leonardo de Melo Versieux (Federal University of Rio Grande do Norte, UFRN), and a voucher specimen (UFRN10854) was deposited in the UFRN herbarium. Aqueous infusions were prepared using 50 g of leaves in 1000 mL of boiling distilled water (100 °C). The vessel was covered with aluminium foil and maintained at boiling temperature for 30 min. The infusion was then filtered through Whatman No. 1 filter paper and freeze-dried. The dried material was subsequently partitioned with ethyl acetate (10 × 100 mL), followed by *n*-butanol (10 × 100 mL). The combined ethyl acetate and butanol fractions were concentrated under reduced pressure using a rotary evaporator prior to analysis.

### 4.3. Fingerprinting by HPLC-ESI-QTOF-MS/MS

High-performance liquid chromatography coupled with mass spectrometry (HPLC–MS) was used for the tentative identification of compounds in the aqueous infusion of *T. esculenta*. Analyses were performed on an Agilent Technologies system comprising an HPLC (1200 Series) coupled to a quadrupole time-of-flight (Q-TOF) mass spectrometer equipped with an electrospray ionisation (ESI) source operating in negative mode. The instrument settings were as follows: fragmentor voltage, 110 V; collision-induced dissociation (CID) energies, 10 and 20 V; capillary voltage, 3000 V; skimmer voltage, 65 V; and nozzle voltage, 1000 V. The mass range was *m*/*z* 40–1200. The gas temperature was set at 250 °C, with the sheath gas at 300 °C, a flow rate of 12 L/min, and a nebuliser pressure of 35 psi.

Chromatographic separation was achieved using an RP-18 Zorbax Eclipse Plus column (150 mm × 2.1 mm, 3.1 µm). A gradient elution was applied using water with 0.1% formic acid (A) and acetonitrile with 0.1% formic acid (B) as follows: 0 min, 1% B; 10 min, 20% B; 15 min, 40% B; 17–22 min, 95% B; and 22–30 min, 1% B. The total run time was 30 min, followed by a 5 min post-run. The flow rate was 0.2 mL/min, and the column temperature was maintained at 20 °C. Data acquisition and processing were performed using MassHunter software (version B.10.00).

Compound identification was based on accurate mass measurements, MS/MS fragmentation patterns, and comparison with published literature and publicly available databases, including GNPS and METLIN.

Quantitative analysis of the flavonoids rutin and quercitrin was performed using the same LC–MS method described above for phenolic profiling. All chromatographic and instrumental conditions, including ionisation mode, were identical to those used for qualitative analysis and had previously been validated for phenolic compounds.

Quantification was carried out using external calibration curves constructed with authentic reference standards of rutin and quercitrin (purity > 95%; Sigma-Aldrich, St. Louis, MO, USA). Stock solutions (1 mg/mL) were prepared in methanol and diluted to working concentrations ranging from 0.001 to 0.1 mg/mL. Calibration curves were generated by plotting peak area against concentration, and linear regression confirmed excellent linearity across the range (R^2^ > 0.99).

Detection was performed in negative electrospray ionisation (ESI−) mode using extracted ion chromatograms (EICs) corresponding to the deprotonated molecular ions [M − H]^−^. Quantification was based on integrated peak areas, ensuring selectivity and reproducibility.

Limits of detection (LOD) and quantification (LOQ) were calculated based on signal-to-noise ratios. The method demonstrated sufficient sensitivity for both analytes in all samples. Precision was evaluated through replicate analyses, with relative standard deviation (RSD) values within acceptable limits, confirming method robustness.

All analyses were performed in triplicate, and results are expressed as mean values.

### 4.4. High-Speed Counter Current Chromatography (HSCCC) Fractionation

#### 4.4.1. Solvent System

To identify the most suitable solvent system for HSCCC separation, four biphasic mixtures of ethyl acetate, *n*-butanol, and water were evaluated in the following ratios: (a) 4:1:5, (b) 4.5:0.5:5, (c) 5:0.5:4.5, and (d) 5:1:4. The optimal distribution of compounds between phases was achieved with the system ethyl acetate:*n*-butanol:water (4.5:0.5:5), which was therefore selected for further use.

For TLC analysis, ethyl acetate:formic acid:acetic acid:water (10:1.1:1.1:0.5) was used as the mobile phase. Approximately 2 mg of each ethyl acetate and *n*-butanol extract was dissolved in the respective solvent systems and applied to silica gel TLC plates. Plates were developed, sprayed with NP/PEG reagent, and visualised under UV light (365 nm) according to previously described procedures.

#### 4.4.2. HSCCC Separation Procedure

The biphasic solvent system was prepared in a separatory funnel, thoroughly mixed, and allowed to equilibrate until complete phase separation. Each phase was collected separately and degassed by sonication for 5 min. For sample preparation, 0.2 g of ethyl acetate extract was dissolved in a 1:1 (*v*/*v*) mixture of the equilibrated upper and lower phases. Reverse-phase HSCCC was performed with the organic phase as the stationary phase and the aqueous phase as the mobile phase. The column was first filled with the stationary phase. The mobile phase was then pumped at 2.0 mL/min while the column rotated at 860 rpm at ambient temperature (25 °C). Once hydrodynamic equilibrium was achieved, the sample was injected. Fractions of 4 mL were collected every 2 min, yielding a total of 110 fractions (60 during elution and 50 during extrusion).

#### 4.4.3. Purification of Isolated Compounds

Fractions were monitored by TLC, and those with similar Rf values were combined. The pooled fractions were further purified by size-exclusion chromatography on a Sephadex LH-20 column, eluted with methanol at a flow rate of 0.5–1.0 mL/min. This procedure enabled the isolation of rutin.

#### 4.4.4. NMR Identification

Structural elucidation of rutin and quercitrin was performed using a Bruker Avance 400 MHz NMR spectrometer. ^1^H-NMR spectra were recorded at 400 MHz and ^13^C-NMR spectra at 100 MHz. Two-dimensional experiments (COSY, HMBC, and HSQC) were also acquired. Chemical shifts are reported in ppm and coupling constants (J) in Hz.4.4.5. HPLC analysis.

Further characterisation was carried out using a Waters Alliance 2695 HPLC system equipped with a dual-wavelength UV detector. Separation was achieved on a LiChrospher RP-18 column (25 cm × 4.6 mm). The mobile phase consisted of solvent A (0.25% phosphoric acid in water) and solvent B (methanol), using the following gradient: 0–5 min, 40% B; 5–10 min, 55% B; 10–15 min, 65% B; 15–20 min, 50% B; 20–25 min, 30% B; and 25–30 min, 40% B. The flow rate was 1.0 mL/min, the injection volume was 10 µL, and detection was performed at 254 and 280 nm.

### 4.5. Antioxidant Activity

A range of antioxidant assays was used to evaluate radical-scavenging activity, reducing capacity, and metal-chelating ability. All samples were tested at a concentration of 100 µg/mL.

#### 4.5.1. Total Phenolic Content

Total phenolic content was determined using the Folin–Ciocalteu method [34], with gallic acid (0.1–5 mg/mL) as the standard. Absorbance was measured at 760 nm, and results were expressed as mg gallic acid equivalents (GAE) per mg of extract.

#### 4.5.2. Total Antioxidant Capacity (TAC)

Total antioxidant capacity was assessed based on the reduction of Mo(VI) to Mo(V) [35]. Samples were incubated with reagent solution at 100 °C for 90 min, cooled, and absorbance was measured at 695 nm. Results were expressed relative to ascorbic acid.

#### 4.5.3. DPPH Radical Scavenging

The DPPH assay was performed in 96-well plates. After 30 min incubation in the dark, absorbance was measured at 517 nm, and results were expressed as percentage radical scavenging activity [17].

#### 4.5.4. Copper Chelation

Copper-chelating activity was evaluated using pyrocatechol violet. Absorbance was measured at 632 nm, and results were expressed as percentage chelation [17].

### 4.6. Antimicrobial Activity

#### 4.6.1. Medium and Bacterial Strains

The antibiotics used in this study were chloramphenicol, kanamycin, and vancomycin (Sigma-Aldrich, Arklow, Ireland). The bacterial strains tested were:

*Staphylococcus aureus* NCTC 6571
*Escherichia coli* MG1655
*Pseudomonas aeruginosa* PAO1
*Listeria monocytogenes*
*Salmonella enterica* serovar Typhimurium UK1

#### 4.6.2. Minimum Inhibitory Concentration (MIC) and Minimum Bactericidal Concentration (MBC) Assay

All strains were revived from −80 °C stocks and subcultured prior to experimentation. Minimum inhibitory concentration (MIC) and minimum bactericidal concentration (MBC) assays were performed using broth microdilution in accordance with CLSI (2020) guidelines. Growth kinetics were monitored at 600 nm over 24 h. Samples were prepared in dimethyl sulfoxide (DMSO) and serially diluted to final concentrations ranging from 50 to 0.781 µg/mL.

#### 4.6.3. Biofilm Assay

Following MIC determination, wells were washed with saline and stained with crystal violet. After drying, the bound dye was solubilised using 33% acetic acid, and absorbance was measured at 570 nm. All experiments were performed in quadruplicate. Biofilm quantification followed the method described by Shukla and Rao [36].

### 4.7. Molecular Docking

Molecular docking studies were conducted using UCSF Chimera (v1.17.3) (available at https://www.cgl.ucsf.edu/chimera/ (accessed on 25 December 2025)) with AutoDock Vina Plug in, version 1.1.2, integrated into the execution interface and available from its official distribution repository (https://github.com/ccsb-scripps/AutoDock-Vina (accessed on 25 December 2025)). Protein structures were retrieved from the Protein Data Bank, and ligand structures were obtained from PubChem (Table 3). Docking parameters were set as follows: exhaustiveness, 8; number of binding modes, 9; and energy range, 3 kcal/mol. The best-scoring conformations were selected based on binding energy, and hydrogen bond interactions were analysed using relaxed geometric criteria.

### 4.8. Macrophage Cell Culture and NF-κB Reporter Assay

RAW 264.7 macrophages (RAW-Blue™; catalogue no. raw-sp, InvivoGen, Toulouse, France) were used to evaluate anti-inflammatory activity via modulation of NF-κB activation. Cells were seeded in 96-well plates at a density of 1 × 10^4^ cells per well in Dulbecco’s Modified Eagle Medium (DMEM; Gibco, Grand Island, NY, USA) supplemented with 10% foetal bovine serum (FBS) and incubated at 37 °C in a humidified atmosphere with 5% CO_2_. After 24 h, the medium was replaced with serum-free DMEM, and incubation continued for a further 24 h.

Three treatment conditions were applied: (i) co-treatment with extract and lipopolysaccharide (LPS); (ii) pre-treatment with extract for 5 h prior to LPS exposure; and (iii) pre-treatment for 10 h before LPS addition. Extracts were tested at concentrations of 250, 500, and 750 µg/mL. After treatment, cells were incubated for 20 h.

For NF-κB quantification, 20 µL of culture supernatant was transferred to a fresh 96-well plate and mixed with 180 µL of QUANTI-Blue™ reagent (InvivoGen, Toulouse, France). Plates were incubated at 37 °C for 45 min, and absorbance was measured at 570 nm using a BioTek Epoch microplate reader (BioTek Instruments, Santa Clara, CA, USA). Positive and negative controls were included in all experiments, and results were normalised to the negative control.

## 5. Conclusions

The present study expands current phytochemical knowledge of *T. esculenta* leaves through the characterisation of flavonol glycosides and related phenolic compounds, including rutin and quercitrin. Fractionation and isolation approaches revealed the distribution of antioxidant and antibacterial activities among enriched fractions and purified constituents, highlighting flavonoids as key contributors to the biological properties of this species.

The antioxidant activity observed in the crude extract and enriched fractions supports the role of phenolic constituents in redox-related mechanisms. Furthermore, modulation of inflammatory signalling in macrophage-based assays suggests that these secondary metabolites may influence cellular pathways associated with immune responses. The inhibition of *Staphylococcus aureus* growth and biofilm formation, together with molecular docking analyses targeting adhesion-related protein domains, further indicates that flavonol-rich fractions may interfere with bacterial proliferation and surface-associated processes.

Despite these findings, several limitations should be acknowledged. First, the study relies primarily on in vitro assays, which do not fully replicate the complexity of in vivo physiological conditions, including metabolism, bioavailability, and compound–matrix interactions. Consequently, the biological relevance of the observed effects may differ under systemic exposure. Second, although LC–MS analysis enabled the identification of the major phenolic constituents, comprehensive structural elucidation and absolute quantification of all detected compounds were not undertaken. Third, while molecular docking provided valuable insights into potential ligand–target interactions, these predictions remain computational and require experimental validation to confirm binding affinity and biological relevance. In addition, potential synergistic or antagonistic interactions among phytochemicals were not systematically investigated, despite evidence that such interactions may significantly influence the activity of crude extracts.

Future studies should therefore address these limitations. In vivo investigations are required to confirm the biological activities observed in vitro and to evaluate pharmacokinetics, safety, and effective dosage ranges. Mechanistic studies focusing on specific molecular targets, including NF-κB, MAPK, and quorum-sensing regulatory pathways, would further elucidate the modes of action of the bioactive fractions. Moreover, transcriptomic and proteomic approaches could provide a more comprehensive understanding of the cellular responses induced by *T. esculenta* extracts.

From an applied perspective, the development of standardised extracts enriched in flavonoid glycosides may offer promising opportunities for the formulation of phytotherapeutic products and functional ingredients. However, variation in plant material arising from environmental and geographical factors must be considered, highlighting the need for cultivation standardisation and robust quality-control strategies. Ultimately, the integration of phytochemical, biological, and clinical evidence will be essential for validating the therapeutic potential of *T. esculenta* and translating these findings into safe and effective applications.

## Figures and Tables

**Table 1:** The list of assigned low-molecular components in the *Talisia esculenta* extract.

No.	Ion	Rt (min)	Formula	*m/z* (Calc.)	*m/z* (Exp.)	Δ (ppm)	RDB	MS/MS Fragments	Compound
1	[M − H]^−^	2.301	C_6_H_8_O_7_	191.0197	191.0182	7.95	3	173.0076 129.0173 111.0073 87.0075	Isocitric acid
2	[M − H]^−^	3.257	C_6_H_8_O_7_	191.0197	191.0213	−8.2	3	173.0064 129.0185 111.0086 87.0090	Citric acid
3	[M − H]^−^	4.158	C_13_H_16_O_9_	315.0722	315.0739	−4.25	6	272.0054 233.0776 180.0431 152.0098 108.0204	Protocatechoylglucose
4	[M − H]^−^	11.029	C_13_H_16_O_10_	331.0671	331.0659	3.52	6	313.0592 168.0023 125.0223	Glucogallic acid
5	[M − H]^−^	11.729	C_13_H_16_O_9_	315.0722	315.0735	−4.25	6	233.0776 180.0431 152.0098 108.0204	Protocatechuic acid glucoside
6	[M − H]^−^	12.68	C_7_H_6_O_4_	153.0187	153.0180	8.65	5	-	Protocatechuic acid
7	[M − H]^−^	13.530	C_15_H_16_O_10_	355.0671	355.0647	6.66	8	209.0231 191.0172 171.0412	Caffeoyl-glucuronide isomer 1
8	[M − H]^−^	13.714	C_22_H_22_O_16_	541.0835	541.0816	3.52	12	387.0614 153.0186	Protocatechuic acid hexoside derivative
9	[M − H]^−^	14.681	C_15_H_16_O_10_	355.0671	355.0669	0.48	8	209.0308 191.0179 180.0316 163.0420 133.0135	Caffeoyl-glucuronide isomer 2
10	[M − H]^−^	15.365	C_30_H_26_O_12_	577.1351	577.1365	−2.34	18	425.0862 407.0778 289.0752 125.0231	Procyanidin B isomer
11	[M − H]^−^	15.848	C_15_H_14_O_6_	289.0674	289.0726	−2.89	9	245.0811 203.0639 123.0437	Catechin
12	[M − H]^−^	16.3	C_27_H_28_O_5_	431.1864	431.1869	−3.24	14	385.1808 223.1264 153.0919	Dimethoxy-dimethyl- (methylbutenyl)-phenyl- pyrano-benzopyranone
13	[M − H]^−^	16.515	C_30_H_26_O_12_	577.1351	577.1348	0.61	18	451.0983 425.0859 407.0741 289.0722 125.0247	Procyanidin B isomer
14	[M − H]^−^	17.350	C_15_H_14_O_6_	289.0674	289.0721	−1.17	9	245.0825 203.0692 137.0212 123.0445	Epicatechin
15	[M − H]^−^	18.067	C_27_H_30_O_16_	609.1461	609.1471	−1.63	13	446.0896 283.0248 255.0285	Luteolin dihexoside
16	[M − H]^−^	19.017	C_27_H_30_O_16_	609.1461	609.1480	−3.1	13	300.0276 271.0255 255.0301 178.9980	Rutin *
17	[M − H]^−^	19.818	C_27_H_30_O_15_	593.1512	593.1513	−0.18	13	285.0393 255.0291	Luteolin hexoside-deoxyhexoside
18	[M − H]^−^	20.352	C_21_H_20_O_11_	447.0933	447.0923	2.2	12	300.0257 255.0277 227.0340 151.0028	Quercitrin **
19	[M − H]^−^	20.985	C_28_H_32_O_16_	623.1618	623.1644	−4.23	13	314.0443 299.0193 271.0277	Isorhamnetin hexoside-deoxyhexoside
20	[M − H]^−^	21.010	C_28_H_32_O_16_	623.1618	623.1610	1.22	13	314.0439 299.0201 271.0238 165.0188	Methoxy-quercetin-*O*-hexoside-deoxyhexoside
21	[M − H]^−^	21.019	C_21_H_20_O_10_	431.0984	431.0990	−1.46	12	284.0331 255.0306 227.0410 183.0394	Luteolin hexoside

(Ion—the type of ionisation, Rt—retention time, calc.—calculated, exp.—experimental, Δ—error of *m/z* measurement, RDB—the number of rings and double bonds, *—compounds identified by NMR method). ** Compounds isolated by high-speed countercurrent chromatography (HSCCC).

**Table 2:** Antimicrobial activity of *T. esculenta* extracts against *Staphylococcus aureus* NCTC 6571.

Extracts	MIC (μg/mL)	MBC (μg/mL)
PT aqueous infusion (PT AI)	12.5	12.5
PT after extraction (PT AE)	39	39
PT ethyl acetate (PT EA)	25	25
PT *n*-Butanol (PT B)	12.5	12.5
Rutin (R)	17	17
Quercitrin (Q)	11.6	11.6

PT = Partition; MIC = minimum inhibitory concentration; MBC = minimum bactericidal concentration.

**Table 3:** Molecular Docking Parameters for Each Protein and Ligand.

Target Protein	Ligand	Grid Box Center (x, y, z)	Grid Box Size (x, y, z)	Exhaustiveness	Number of Modes	Energy Range (kcal/mol)
Surface Protein G (PDB: 3TIP)	Quercitrin	−20.22, 13.24, 47.95	83.39, 13.30, 75.82	8	9	3
Surface Protein G (PDB: 3TIQ)	Quercitrin	−5.68, −31.30, 17.88	73.52, 53.59, 220.86	8	9	3
Surface Protein G (PDB: 3TIP)	Rutin	−24.82, 18.72, 35.48	78.08, 19.05, 75.05	8	9	3
Surface Protein G (PDB: 3TIQ)	Rutin	−23.31, 12.29, 29.52	75.11, 111.90, 218.55	8	9	3
Biofilm-associated Surface Protein (PDB: 7C7U)	Quercitrin	13.75, −23.63, 186.95	47.90, 45.13, 108.79	8	9	3
Biofilm-associated Surface Protein (PDB: 7C7U)	Rutin	10.93, −14.79, 179.54	54.64, 71.32, 129.51	8	9	3
Clumping Factor B (PDB: 4F1Z)	Quercitrin	10.59, −32.45, 10.82	53.09, 41.58, 37.31	8	9	3
Clumping Factor B (PDB: 4F1Z)	Rutin	17.90, −30.54, 13.77	72.15, 45.27, 42.69	8	9	3

## Data Availability

The data is presented in the article and Supplementary Materials. Any other questions can be directed to the corresponding author.

## Supplementary Materials

The following supporting information can be downloaded at: https://www.mdpi.com/article/10.3390/plants15142229/s1, Table S1: MS/MS spectra of compounds identified with the use of HPLC-MS fingerprinting. Table S2: The LOD and LOQ values of the determined rutin and quercitrin in the extract. Figure S1: The MS chromatogram (TIC) of the total extract–at the top, the extracted ion chromatogram (EIC) of rutin with its peak at 19.1 min–in the middle, and the extracted ion chromatogram of quercitrin peak at 20.352 min, recorded in the negative ion mode. Figure S2: The TIC mass chromatogram of *Talisia esculenta* recorded in the positive and negative ion mode. Figure S3: Predicted binding modes of quercitrin and rutin with *Staphylococcus aureus* surface proteins. Docking poses of quercitrin (left panels) and rutin (right panels) are shown bound to the target proteins, displayed as light-blue ribbon structures. (A–D) Surface Protein G (PDB entries 3TIP and 3TIQ). (E,F) Biofilm-associated Surface Protein (PDB: 7C7U). (G–I) Clumping Factor B (PDB: 4F1Z), shown in complex with a keratin-derived peptide (green). The ligands are displayed as sticks and labelled accordingly. The panels show the location of the ligands within the predicted binding sites, indicating potential interference with host–protein interactions, particularly in Clumping Factor B, where the flavonoids bind close to the keratin-binding interface. Figure S4: Molecular representation of key hydrogen-bond interactions between flavonoids and *Staphylococcus aureus* surface proteins. The figure shows selected hydrogen bonds identified in the most favourable docking poses (Model 3.1). Proteins are displayed as light-blue ribbons, ligands as green sticks, and interacting residues as sticks coloured by atom type. Distances are shown in angstroms (Å). (A) Quercitrin interacting with LYS513.A and THR516.A in Surface Protein G (PDB: 3TIP). (B) Quercitrin forming hydrogen bonds with PHE510.A, ASN598.A and GLU509.A in Surface Protein G (PDB: 3TIQ). (C) Quercitrin forming a hydrogen bond with THR703.B in the Biofilm-associated Surface Protein (PDB: 7C7U). (D) Rutin interacting with GLY524.A in Surface Protein G (PDB: 3TIP). (E) Rutin forming hydrogen bonds with PHE510.A and SER610.B in Surface Protein G (PDB: 3TIQ). (F) Rutin forming three hydrogen bonds with ARG693.B in the Biofilm-associated Surface Protein (PDB: 7C7U). (G) Rutin forming hydrogen bonds with ARG331.A and TYR273.A in Clumping Factor B (PDB: 4F1Z), close to the keratin-binding region (shown in green). Figure S5: In vitro assays using RAW 264.7 macrophages treated with *Talisia esculenta* extracts. (A) Effect on NF-κB expression when the samples and lipopolysaccharide (LPS) were added simultaneously. (B) Effect on NF-κB expression when the samples were added 5 h before LPS. (C) Effect on NF-κB expression when the samples were added 10 h before LPS. The y-axis shows the percentage of NF-κB expression relative to the positive control (PC; cells treated with LPS), which was set at 100%. Samples were tested at concentrations of 500 and 750 µg/mL. Negative control (NC) consisted of RAW 264.7 macrophages cultured in medium only, while PC consisted of macrophages treated with LPS in culture medium. AI = water infusion; EA = ethyl acetate fraction; B = *n*-butanol fraction; AE = residual extract obtained after partitioning; rutin and quercitrin = isolated compounds. Experiments were performed in triplicate. Statistical analysis was carried out using Tukey’s test, with significance indicated as *p* < 0.05 (*), *p* < 0.01 (**), *p* **<** 0.001 (***) and *p* < 0.0001 (****).
