# Supplementary material for: Phytochemical Characterization and High-Speed Countercurrent Chromatography-Assisted Isolation of Flavonoids from Leaves of Talisia esculenta Radlk
Source: Plants (Basel). 2026 Jul 21;15(14):2229. doi: 10.3390/plants15142229 (PMC13415155; doi:10.3390/plants15142229)
Supplement: Supplementary file 1 [file plants-15-02229-s001.zip › plants-4358245-supplementary.pdf]

Supplementary Material

Table S1. MS/MS spectra of compounds identified with the use of HPLC-MS fingerprinting.

<table>
<tr><th>No.</th><th>Compound</th></tr>
<tr><td>1</td><td>Isocitric acid

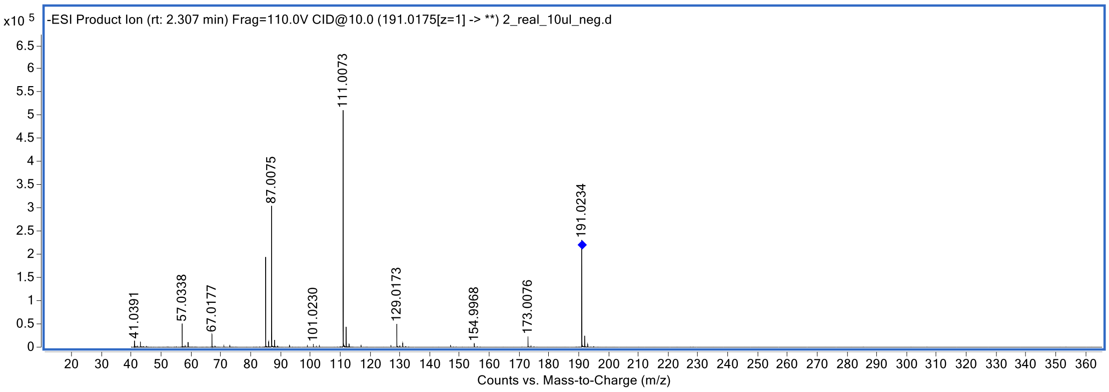

</td></tr>
<tr><td>2</td><td>Citric acid

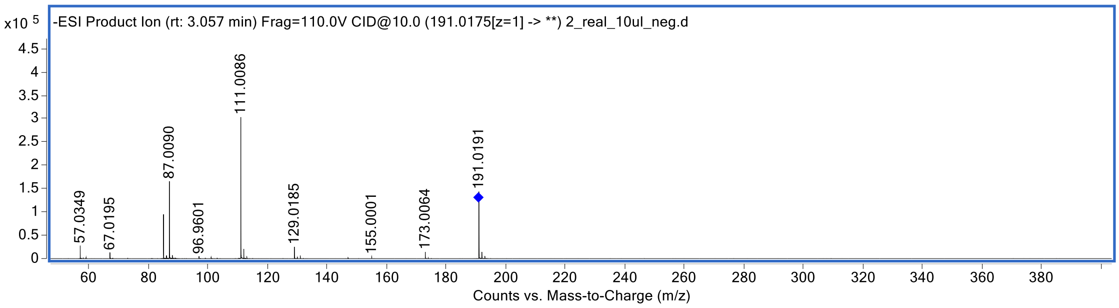

</td></tr>
<tr><td>3</td><td>Protocatechoylglucose

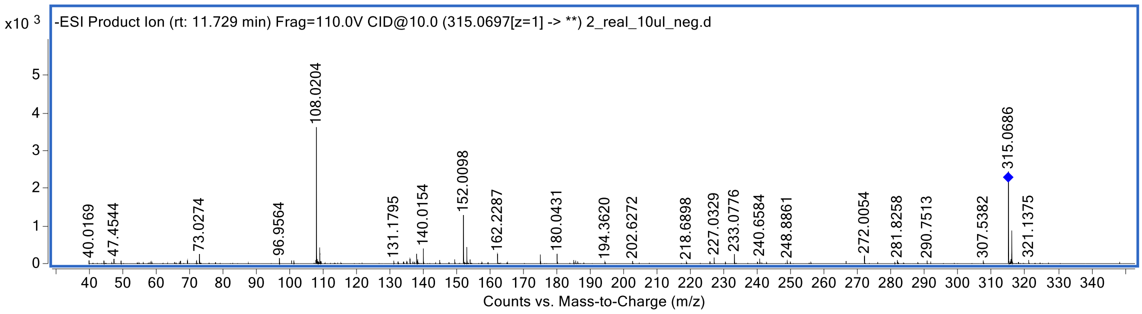

</td></tr>
<tr><td>4</td><td>Glucogallic acid

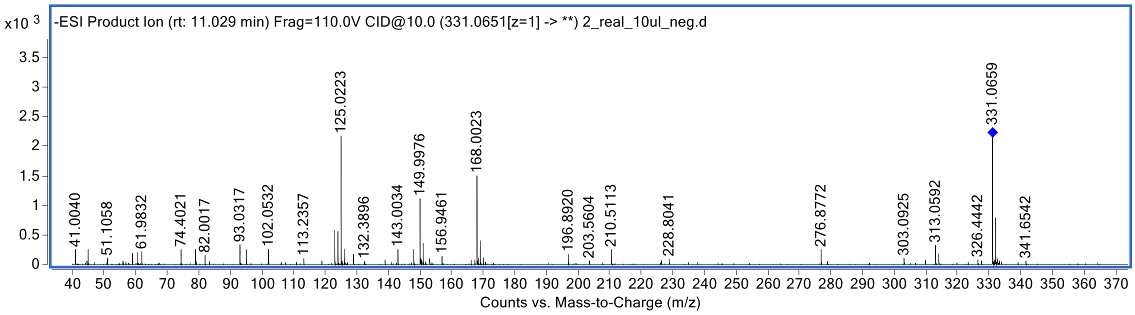

</td></tr>
<tr><td>5</td><td>Protocatechuic acid glucoside</td></tr>
</table>

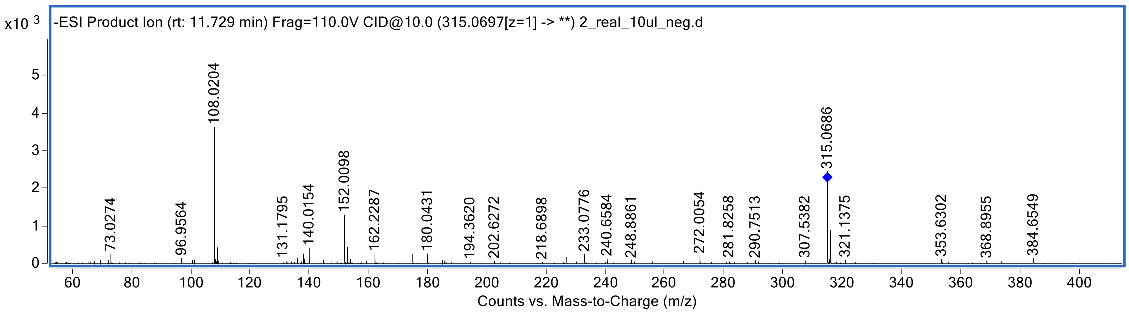

6 Caffeoyl-glucuronide isomer 1

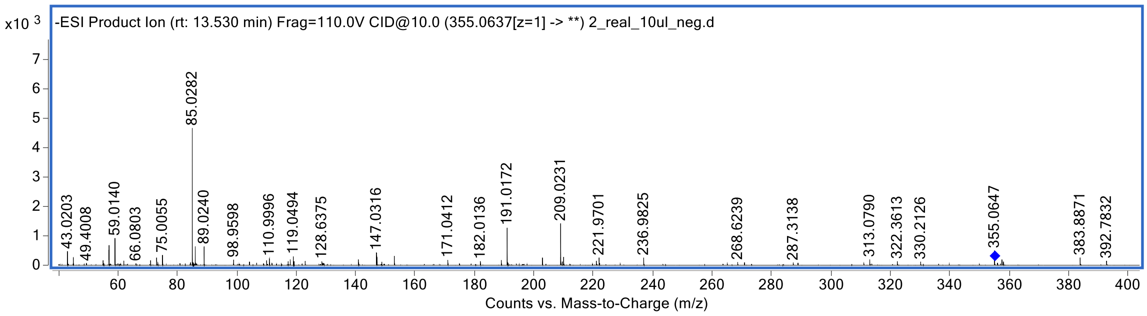

7

Protocatechuic acid hexoside derivative

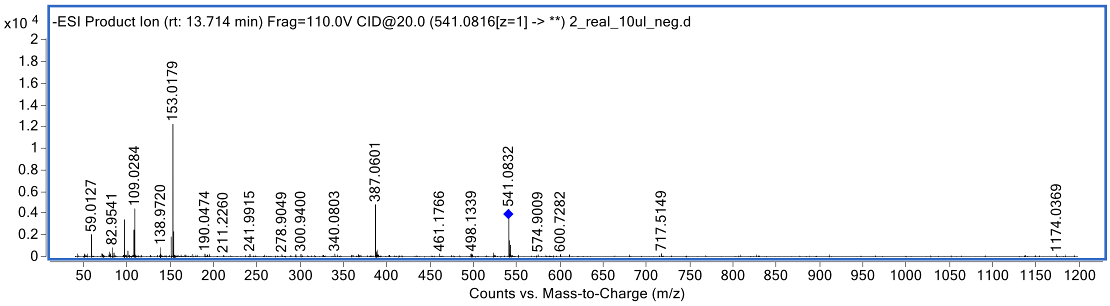

8

Caffeoyl-glucuronide isomer 2

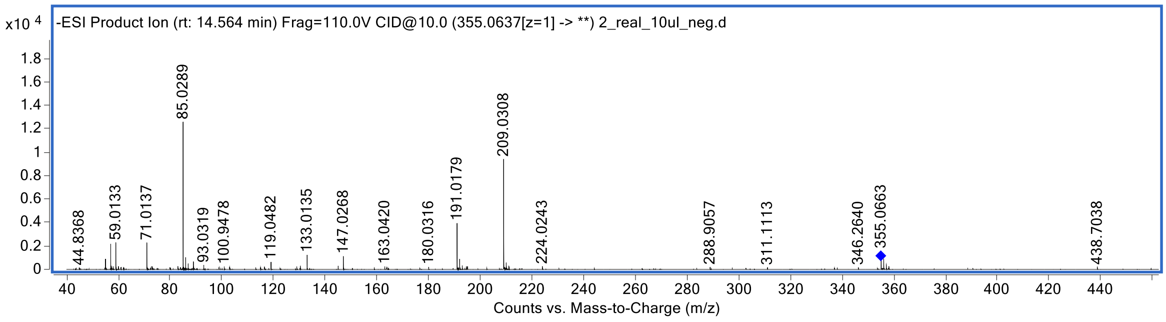

**9**	Procyanidin B isomer
	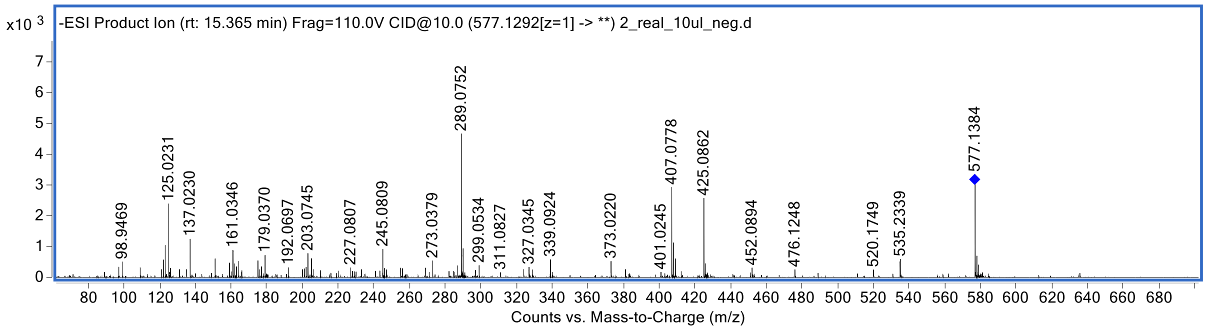
**10**	Catechin
	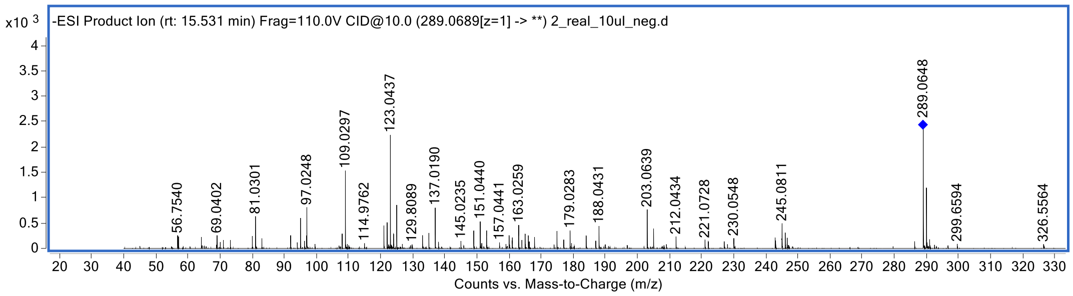
**11**	Dimethoxy-dimethyl-(methylbutenyl)-phenyl-pyrano-benzopyranone
	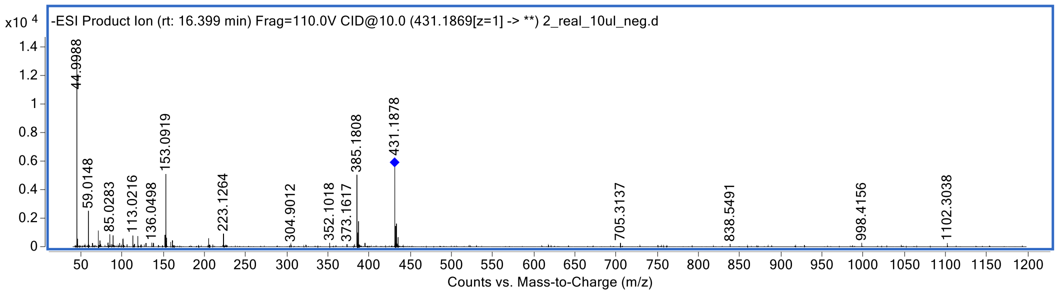
**12**	Procyanidin B isomer
	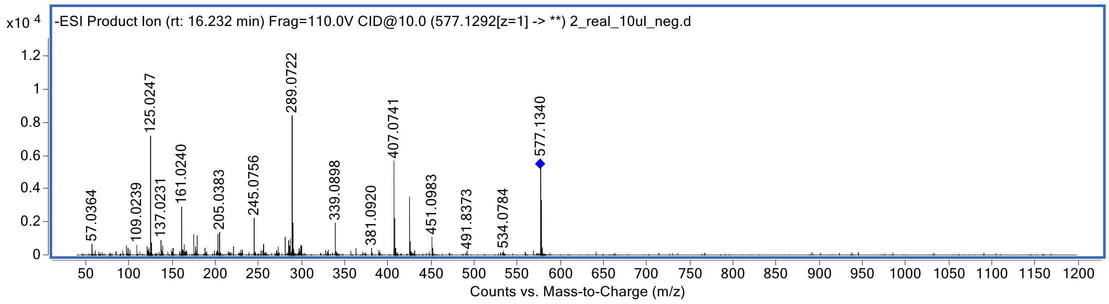
**13**	Epicatechin
	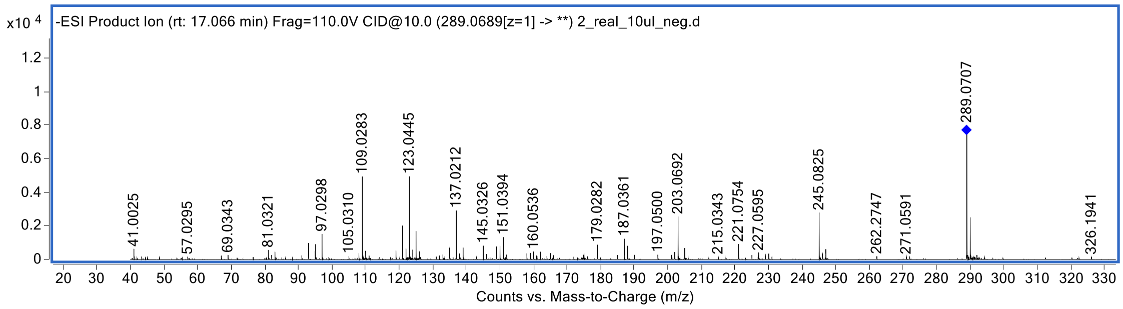

14 Luteolin dihexoside

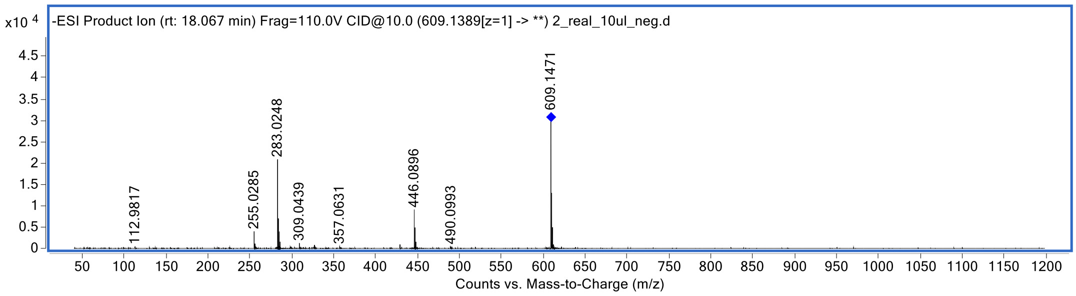

15 Rutin *

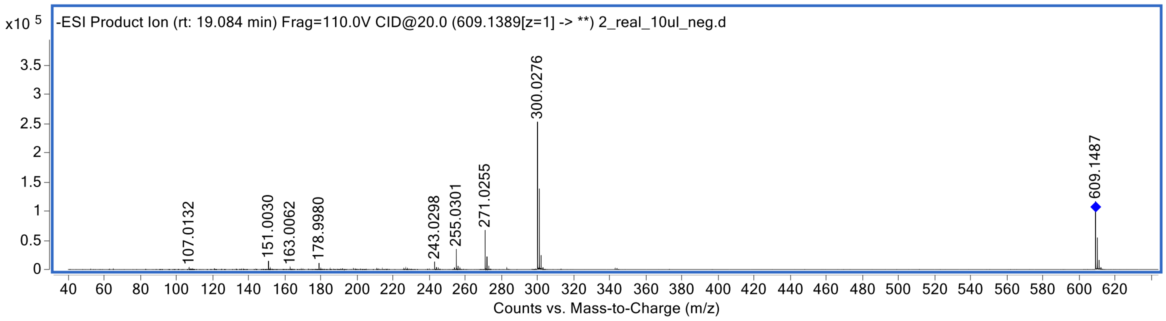

16 Luteolin hexoside-deoxyxhexoside

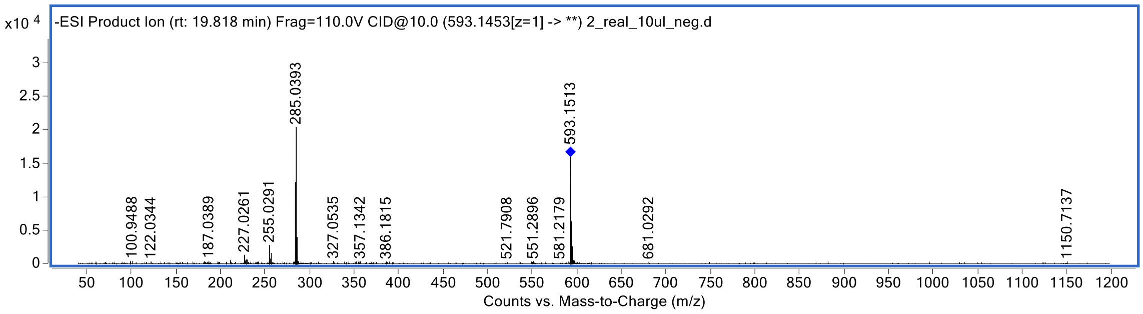

17 Quercitrin *

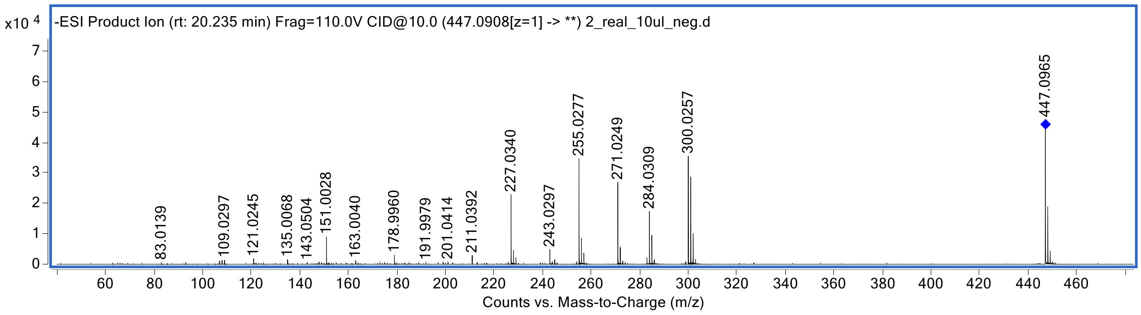

18 Isorhamnetin hexoside-deoxyhexoside

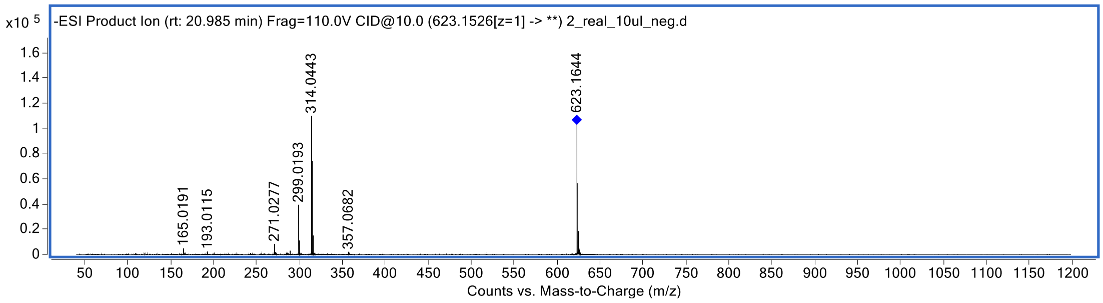

**19**	Methoxy-quercetin-O-hexoside-deoxyhexoside 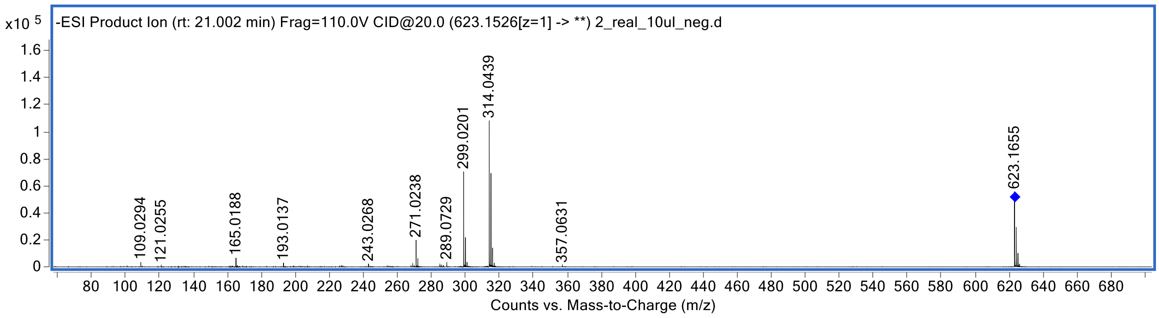
**20**	Luteolin hexoside 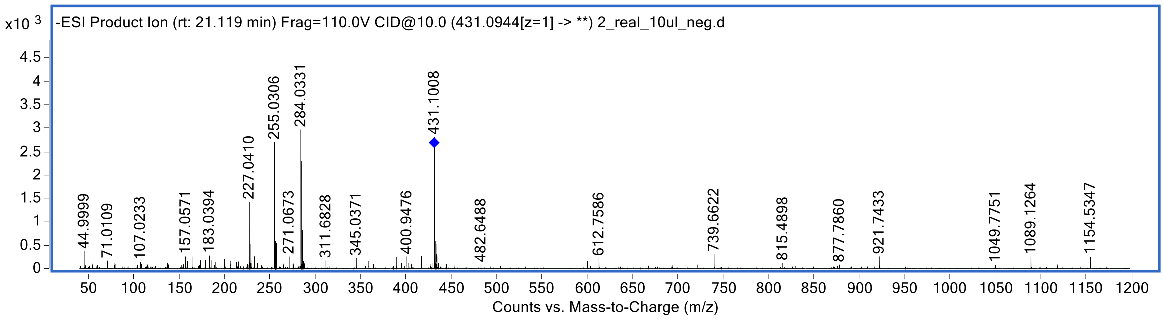

Table S2. The LOD and LOQ values of the determined rutin and quercitrin in the extract.

Reference compound	LOD [mg/mL]	LOQ [mg/mL]
Quercitrin	0.0007	0.00231
Rutin	0.0008	0.00240

LOD = Limit of Detection and LOQ = Limit of quantification

Supplementary Figures

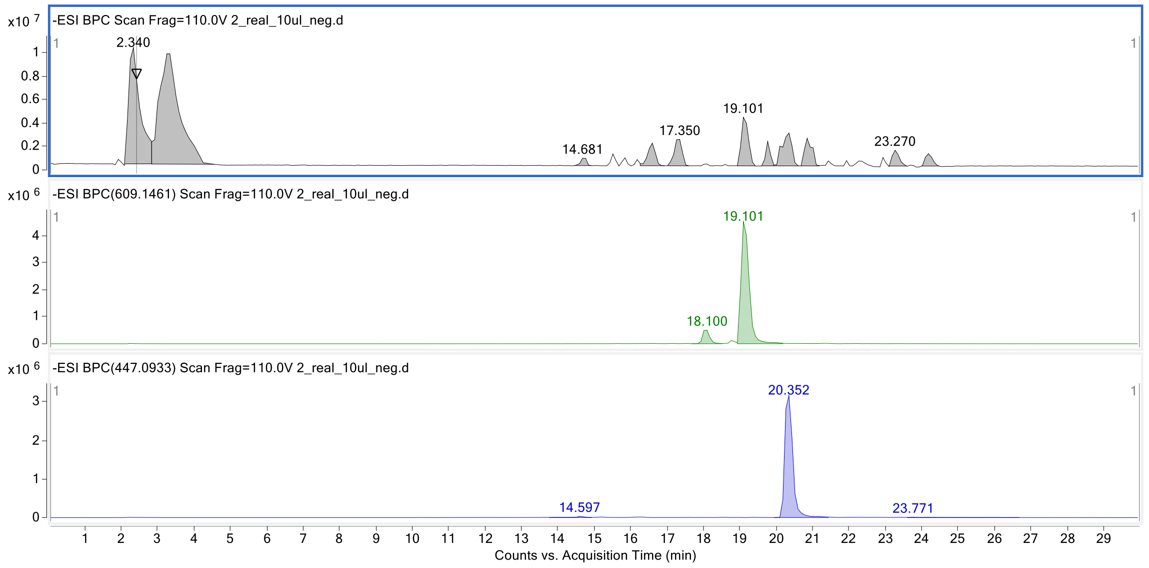

Figure S1. The MS chromatogram (TIC) of the total extract – at the top, the extracted ion chromatogram (EIC) of rutin with its peak at 19.1 min – in the middle, and the extracted ion chromatogram of quercitrin peak at 20.352 min, recorded in the negative ion mode.

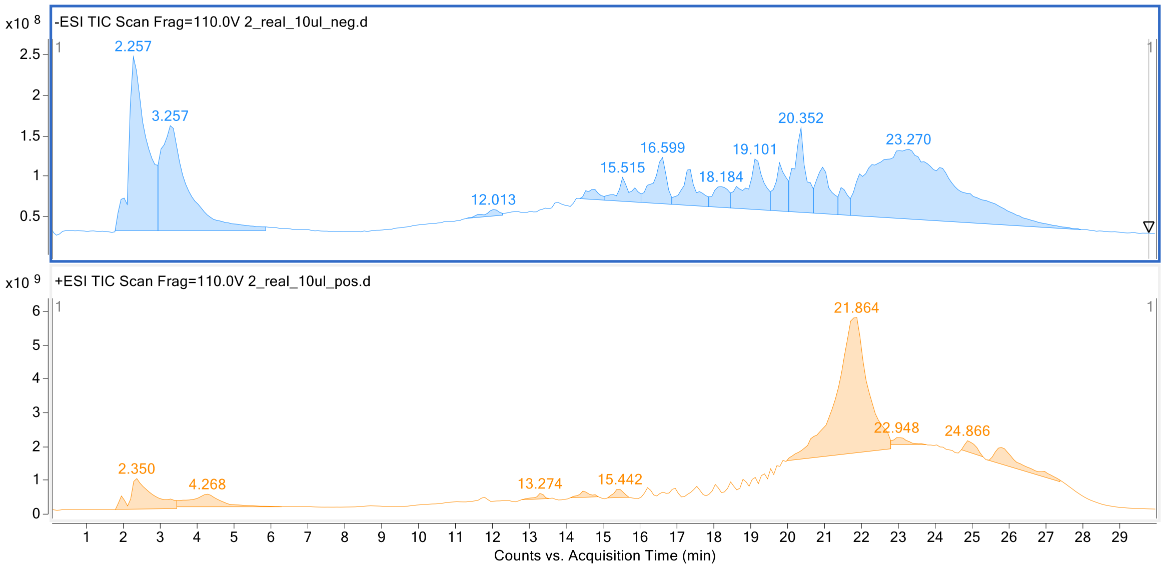

Figure S2. The TIC mass chromatogram of *Talisia esculenta* recorded in the positive and negative ion mode.

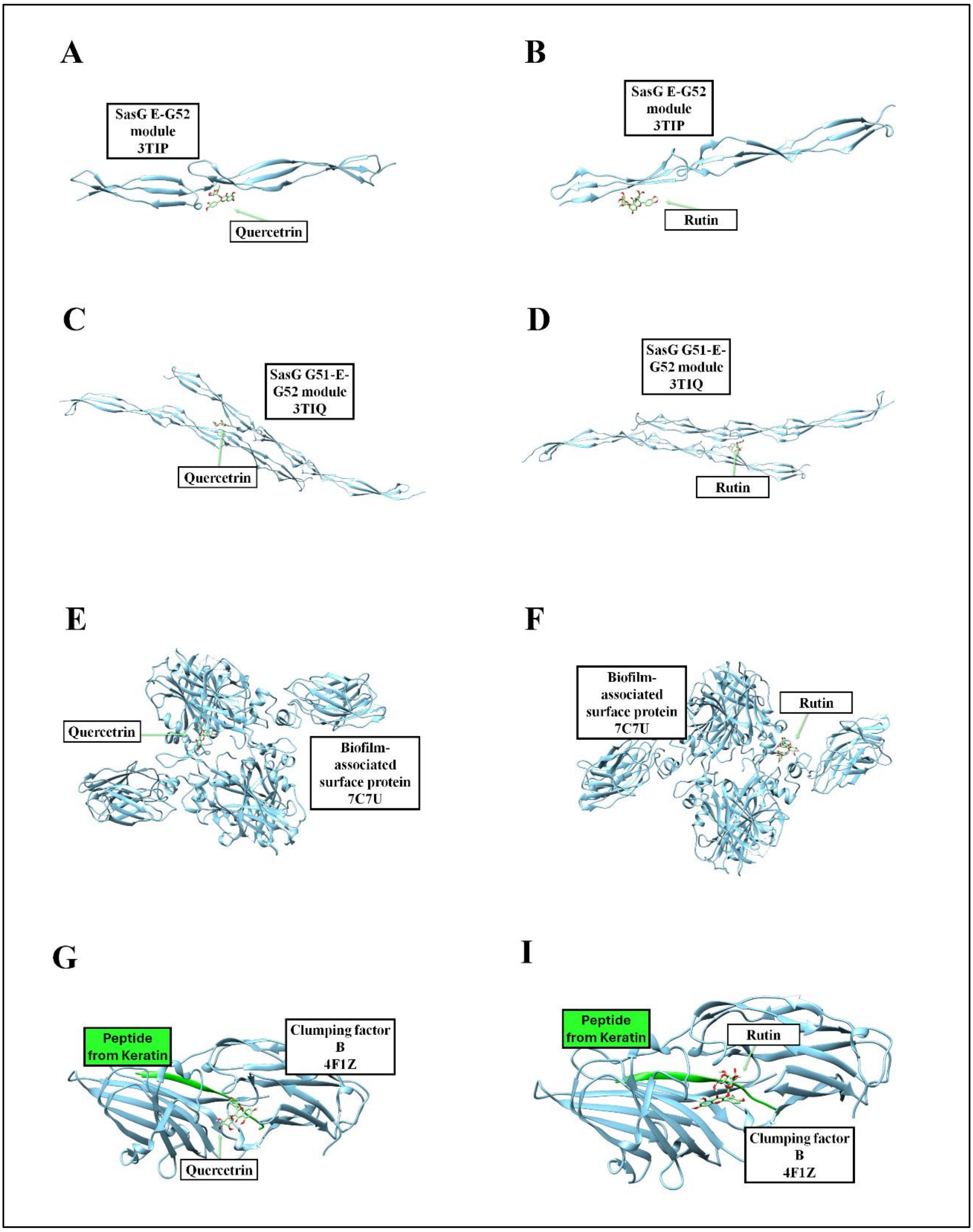

Figure S3. Predicted binding modes of quercitrin and rutin with *Staphylococcus aureus* surface proteins. Docking poses of quercitrin (left panels) and rutin (right panels) are shown bound to the target proteins, displayed as light-blue ribbon structures. (A–D) Surface Protein G (PDB entries 3TIP and 3TIQ). (E–F) Biofilm-associated Surface Protein (PDB: 7C7U). (G–I) Clumping Factor B (PDB: 4F1Z), shown in complex with a keratin-derived peptide (green). The ligands are displayed as sticks and labelled accordingly. The panels show the location of the ligands within the predicted binding sites, indicating potential interference with host–protein interactions, particularly in Clumping Factor B, where the flavonoids bind close to the keratin-binding interface.

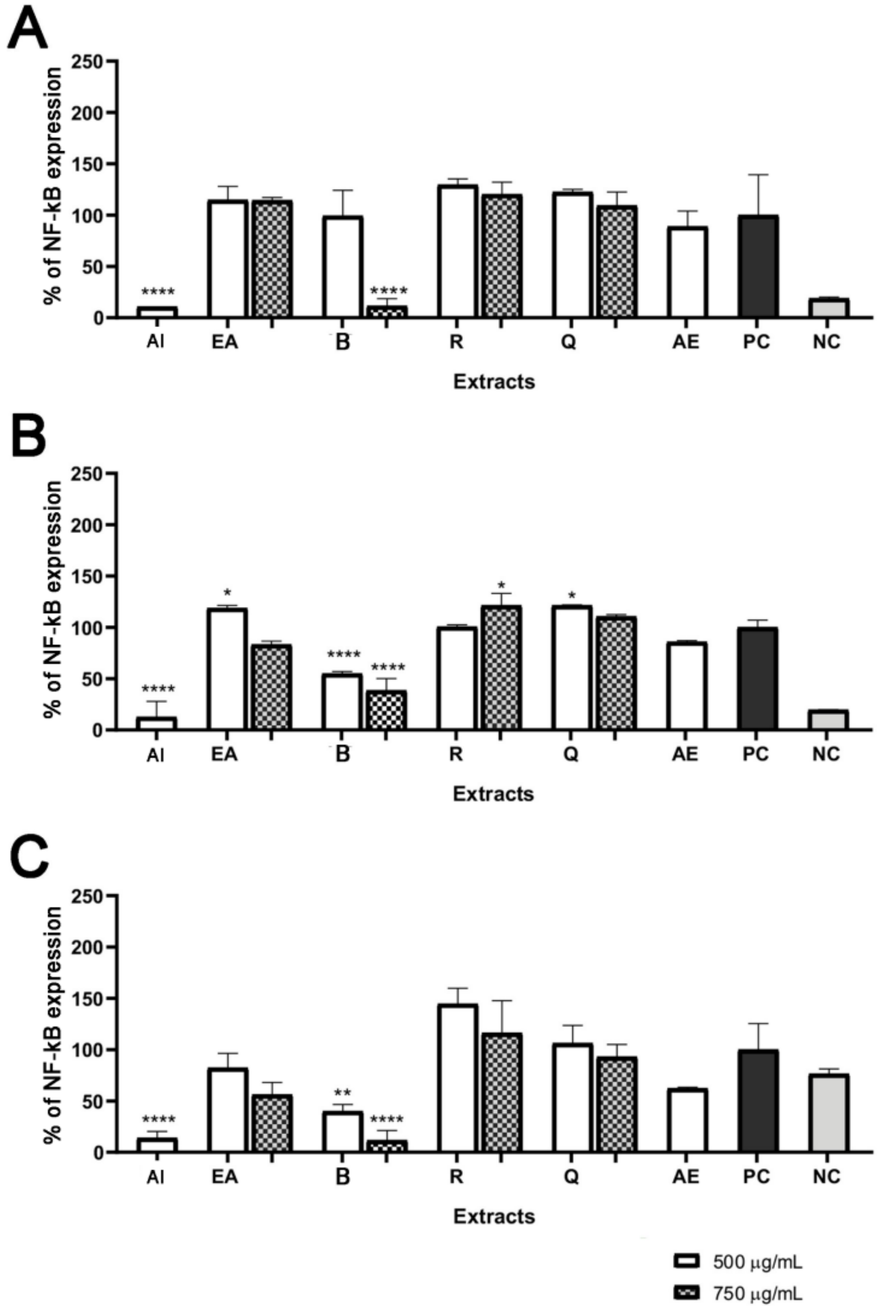

Figure S4. In vitro assays using RAW 264.7 macrophages treated with *Talisia esculenta* extracts. (A) Effect on NF-κB expression when the samples and lipopolysaccharide (LPS) were added simultaneously. (B) Effect on NF-κB expression when the samples were added 5 h before LPS. (C) Effect on NF-κB expression when the samples were added 10 h before LPS. The y-axis shows the percentage of NF-κB expression relative to the positive control (PC; cells treated with LPS), which was set at 100%. Samples were tested at concentrations of 500 and 750 µg/mL. Negative control (NC) consisted of RAW 264.7 macrophages cultured in medium only, while PC consisted of macrophages treated with LPS in culture medium. AI = water infusion; EA = ethyl acetate fraction; B = *n*-butanol fraction; AE = residual extract obtained after partitioning; rutin and quercitrin = isolated compounds. Experiments were performed in triplicate. Statistical analysis was carried out using Tukey's test, with significance indicated as $P < 0.05$ (*), $P < 0.01$ (**), $P < 0.001$ (***) and $P < 0.0001$ (****).

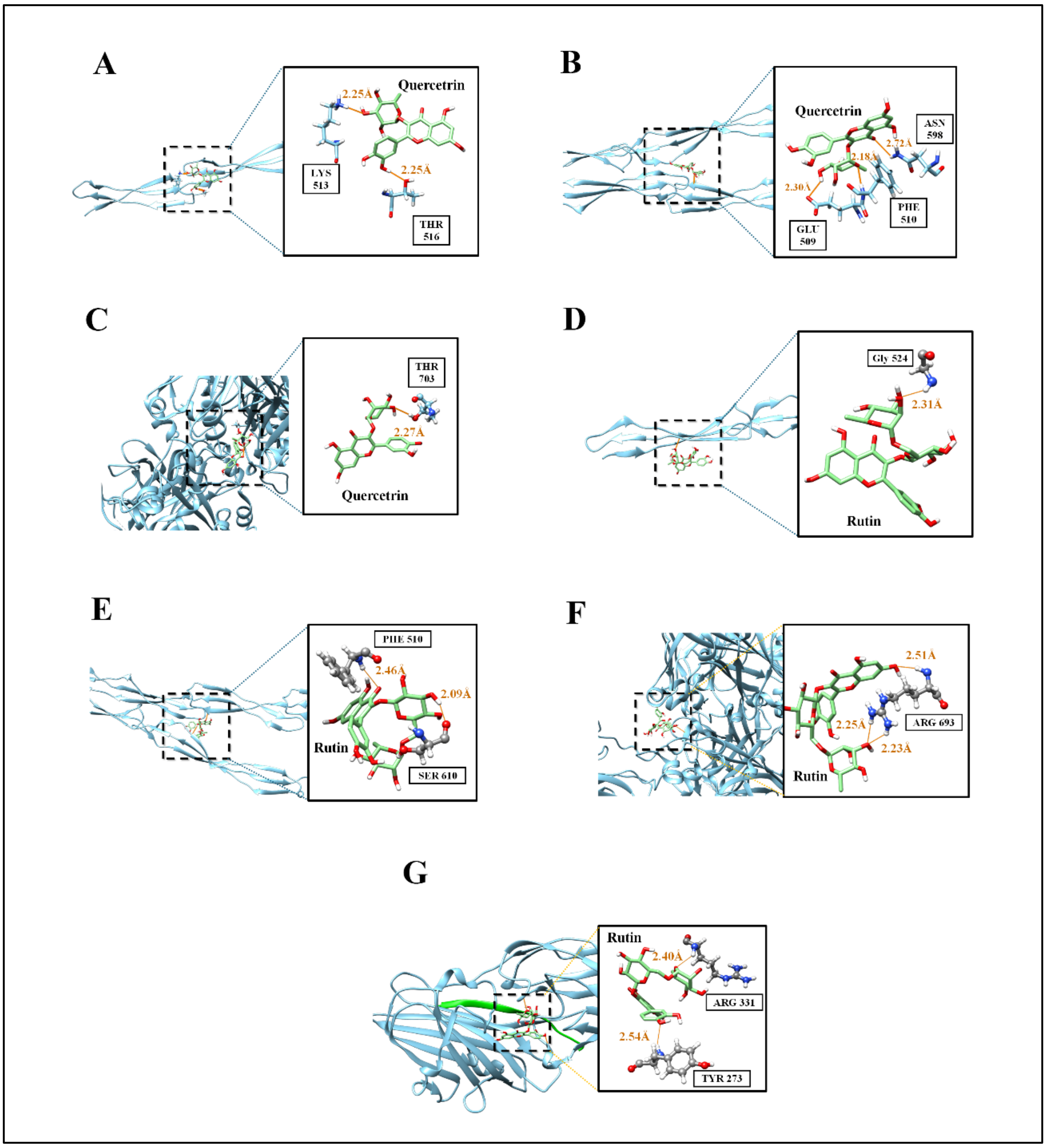

Figure S5. Molecular representation of key hydrogen-bond interactions between flavonoids and *Staphylococcus aureus* surface proteins. The figure shows selected hydrogen bonds identified in the most favourable docking poses (Model 3.1). Proteins are displayed as light-blue ribbons, ligands as green sticks, and interacting residues as sticks coloured by atom type. Distances are shown in angstroms (Å). (A) Quercitrin interacting with LYS513.A and THR516.A in Surface Protein G (PDB: 3TIP). (B) Quercitrin forming hydrogen bonds with PHE510.A, ASN598.A and GLU509.A in Surface Protein G (PDB: 3TIQ). (C) Quercitrin forming a hydrogen bond with THR703.B in the Biofilm-associated Surface Protein (PDB: 7C7U). (D) Rutin interacting with GLY524.A in Surface Protein G (PDB: 3TIP). (E) Rutin forming hydrogen bonds with PHE510.A and SER610.B in Surface Protein G (PDB: 3TIQ). (F) Rutin forming three hydrogen bonds with ARG693.B in the Biofilm-associated Surface Protein (PDB: 7C7U). (G) Rutin forming hydrogen bonds with ARG331.A and TYR273.A in Clumping Factor B (PDB: 4F1Z), close to the keratin-binding region (shown in green).
